# Comprehensive Analysis of mRNA, lncRNA, circRNA, and miRNA Expression Profiles and Their ceRNA Networks in the *Longissimus Dorsi* Muscle of Cattle-Yak and Yak

**DOI:** 10.3389/fgene.2021.772557

**Published:** 2021-12-13

**Authors:** Chun Huang, Fei Ge, Xiaoming Ma, Rongfeng Dai, Renqing Dingkao, Zhuoma Zhaxi, Getu Burenchao, Pengjia Bao, Xiaoyun Wu, Xian Guo, Min Chu, Ping Yan, Chunnian Liang

**Affiliations:** ^1^ Key Laboratory of Yak Breeding Engineering Gansu Province, Lanzhou Institute of Husbandry and Pharmaceutical Science, Chinese Academy of Agricultural Sciences, Lanzhou, China; ^2^ Livestock Institute of Gannan Tibetan Autonomous Prefecture, Hezuo, China; ^3^ Haixi Agricultural and Animal Husbandry Technology Extension Service Center, Qinghai, China

**Keywords:** cattle-yak, *Bos* grunniens, transcriptome, ceRNA, lncRNA, circRNA, skeletal muscle

## Abstract

Cattle-yak, as the hybrid offspring of cattle (*Bos taurus*) and yak (*Bos grunniens*), demonstrates obvious heterosis in production performance. Male hybrid sterility has been focused on for a long time; however, the mRNAs and non-coding RNAs related to muscle development as well as their regulatory networks remain unclear. The phenotypic data showed that the production performance (i.e., body weight, withers height, body length, and chest girth) of cattle-yak was significantly better than that of the yak, and the economic benefits of the cattle-yak were higher under the same feeding conditions. Then, we detected the expression profiles of the *longissimus dorsi* muscle of cattle-yak and yak to systematically reveal the molecular basis using the high-throughput sequencing technology. Here, 7,126 mRNAs, 791 lncRNAs, and 1,057 circRNAs were identified to be differentially expressed between cattle-yaks and yaks in the *longissimus dorsi* muscle. These mRNAs, lncRNA targeted genes, and circRNA host genes were significantly enriched in myoblast differentiation and some signaling pathways related to muscle development (such as HIF-1 signaling pathway and PI3K-Akt signaling pathway). We constructed a competing endogenous RNA (ceRNA) network and found that some non-coding RNAs differentially expressed may be involved in the regulation of muscle traits. Taken together, this study may be used as a reference tool to provide the molecular basis for studying muscle development.

## Introduction

Yak, a special germplasm resource mainly inhabiting the Qinghai-Tibet Plateau, has been optimized for living and a source of living for the local herdsmen. The cattle-yak constitutes the hybrid of cattle (*Bos taurus*) and yak (*Bos grunniens*) exhibiting outstanding hybrid vigor in growth rate, meat performance, plateau adaptability, etc. The meat of cattle-yak is highly enriched in protein but has lower fat than yak, meeting the requirements of a popular healthy and high-quality diet (Song et al., 2019; Wang et al., 2021b). Inhabiting the high-altitude environment, cattle-yak provides a natural green food favored by the consumers. The crossbreeding technology has been widely applied in animal breeding, as well as these hybrid individuals have been farmed for a long time forming gradually new indigenous breeds. Therefore, it is highly significant to understand the mechanisms regulating muscle growth and development of the generated crossbreed. The *longissimus dorsi* (LD) muscle is one of the important representatives of muscle tissues in animals, which is closely related to the individual skeletal muscle growth and development, as well as with the intramuscular fat content, and tenderness. A series of reported myogenic regulatory molecules regulate the myoblast’s proliferation and myophagism, further influencing the growth process, including the identified genes *MYOG* (Rudnicki and Jaenisch, 1995) and *MYF5* (Xu et al., 2019), which are involved in regulating the myoblast differentiation, muscle growth, and meat quality traits in livestock. Previous studies have indicated that *MYOD* as a key regulator of myotube formation promotes the myotube’s differentiation (Tapscott, 2005; Wang et al., 2017). The *CAPN* family are important candidate genes in the growth and degradation of the muscle fibers, and are specifically expressed in the skeletal muscle (Gandolfi et al., 2011).

In fact, the studies indicated that the biological processes were not only regulated by protein-coding RNA—mRNA, as the sequencing technology developed. Non-coding RNAs (ncRNAs), including long non-coding RNA (lncRNA), circular RNA (circRNA), and microRNA (miRNA), are profoundly involved in diverse biological processes and are regulating them by various mechanisms. The lncRNAs universally acknowledged participating in chromatin transcriptional/epigenetic regulation by interacting with the chromatin regulators as “molecular scaffold” or decoys to activate or repress transcription (Caretti et al., 2006; Korostowski et al., 2012). Many lncRNAs have been proven to play a vital role in skeletal muscle development; for example, the lncRNA MAR1 positively correlates with muscle differentiation and growth *in vitro* and *in vivo* (Zhang et al., 2018). The lnc-smad7/miR-125b/*Smad7* (*SMAD* family member 7) and *IGF2* axes are instrumental in myoblast differentiation and regeneration of muscle in two different pig breeds (Song et al., 2018). In addition, continuously growing discoveries have reported the role of novel circRNA in skeletal muscle. CircZfp609 derived from Zinc Finger Protein 609, can inhibit the myogenic differentiation *via* the sponge miR-194-5p in the mouse myoblast cell line C2C12 (Wang et al., 2019b). The circFGFR4 was generated from the fibroblast growth factor receptor 4 (*FGFR4*) and could simulate the bovine primary myoblast’s differentiation through the circFGFR4-miR-107-*WNT3A* axis in cattle (Li et al., 2018). MicroRNA response elements are considered to be “talking mediators” of mRNAs, lncRNAs, and transcribed pseudogenes (Ji et al., 2019), and these response elements have important roles in various biological processes by forming a large number of complex regulatory networks. Numerous reports on the conjoint effect of miRNA and mRNA conjointly on developing skeletal muscle have been proved. *FGFR1*, which could prevent muscle fibrogenesis, is a functional target of miR-214-3p (Arrighi et al., 2021). The miR-183 and miR-96 were found to negatively regulate fat usage in the skeletal muscle *via* targeting *FoxO1* and *PDK4* (Wang et al., 2021a). Zhang et al. (2021) experimentally confirmed that miR-22-3p regulated the *WFIKKN2* gene in adipocyte differentiation in muscle fat metabolism of Yanbian cattle. A targeted relationship between the oar-miR-655-3p and oar-miR-381-5p with *ACSM3* and *ABAT* has been found to have crucial roles in sheep muscle organogenesis, myoblast migration (Sun et al., 2019).

In recent years, with the deepening of the research on the function of miRNAs, a new theory named competing endogenous RNA (ceRNA) has emerged. At the same time, some studies reported that mRNAs, lncRNAs, and circRNAs might regulate the gene function *via* miRNA and act as ceRNAs in various biological processes (Salmena et al., 2011; Yu et al., 2019). In the whole transcriptome, a comprehensive post-transcriptional regulatory network formed by ceRNA activity has greatly widened the cognition of functional genetic information in the genome. There are effective interactions among the lncRNA, circRNA, and mRNA with miRNA, and they can take significant effect in various processes of regulation in animals. LncRNAs act as molecular sponges for the miRNAs that specifically inhibit the target mRNAs so that they can give play to the protection of mRNAs (Li et al., 2019). For instance, a previous study reported that *MAML1* and *MEF2C*, as transcription factors, activate the late-differentiation muscle genes, and linc-MD1 can regulate their expression as a ceRNA by sponging miR-133 and miR-135 (Cesana et al., 2011). LncRNA H19 can regulate muscle differentiation as a molecular sponge for the *LET7* family in the developing embryo and adult muscles (Kallen et al., 2013).

Until now, most studies have been based on focusing on the cattle-yak for exploring the male sterility mechanism and barely referred to the superiority in the growth mechanism. Here, we have measured the growth traits of cattle-yaks and domestic Ashidan yaks under the same feeding and management, and systematically explored the differences of the *longissimus dorsi* muscles for the first time using the whole-transcriptome sequencing. Furthermore, the ceRNA network was constructed to identify the key factors involved in muscle growth and development. This study will thus help in improving yak breeding and provide new ideas for studying the genetic mechanism of muscle growth.

## Materials and Methods

### Ethics Approval

All the animal experiments were approved by Lanzhou Institute of Husbandry and Pharmaceutical Sciences of the Chinese Academy of Agricultural Sciences (CAAS) with the grant number: No. 2019-002. All the slaughter as well as sampling procedures strictly complied with the Guidelines on the Ethical Treatment of Experimental Animals of China.

### Phenotypic Data Collection and Samples Preparation

Thirty cattle-yaks (Aberdeen Angus ♂ × Yak ♀) and 30 yaks were tracked to measure the production performance indices (withers height, body weight, chest girth, and body length) at three stages of growth (i.e., birth, 3 months, and 6 months). Cattle-yaks (*n* = 3, 6 months old) and yaks (*n* = 3, 6 months old) were selected randomly to be slaughtered for *longissimus dorsi* muscle. These samples were collected for transcriptome sequencing and Real-time quantitative polymerase chain reaction analysis (RT-qPCR). All the samples were stored in liquid nitrogen (−80°C) for the subsequent tests.

### RNA Isolation and Illumina Sequencing

The total RNA was isolated with TRIzol (Invitrogen, Carlsbad, CA, United States) following the manufacturer’s instructions, and the concentration and quality of RNA were assessed by 1.5% agarose gel electrophoresis and Thermo Scientific NanoDrop 2000c (ThermoFisher Scientific Inc., Waltham, MA, United States).

Equal quantities of RNA were pooled from each sample. Then, the TruSeq Stranded Total RNA with Ribo-Zero Gold Kit (Illumina, San Diego, CA, United States) was used for digesting the ribosomal RNA (rRNA) in the DNA-free RNA. According to the manufacturer’s instructions, we performed the construction of library preparation with NEB Next Ultra Directional RNA LibraryPrep Kit for Illumina (NEB, Ipswich, MA, United States). The size and purity of libraries were validated by Agilent Technologies 2100 Bioanalyzer (Agilent, Santa Clara, CA). Finally, the samples were sequenced using Illumina HiSeq 2500 Technology (LC Sciences, Houston, TX, United States) with a 150-bp paired-end run.

### Data Preprocessing, Read Mapping, and Transcript Assembly

Raw reads generated during high-throughput sequencing were in fastq format. The raw sequencing dataset supporting the results of cattle-yaks in this study was deposited at NCBI’s Gene Expression Omnibus database (http://www.ncbi.nlm.nih.gov/geo/, accession number: PRJNA753699). The raw sequencing dataset of yaks has been uploaded to the NCBI Database in the previous study (Ma et al., 2020). To get high-quality clean reads that could be used for later analysis, the Trimmomatic software (Bolger et al., 2014) was applied to remove adaptors, low-quality bases, and N-bases.

The remaining high-quality cleaned reads of each sample were aligned to the yak reference genome (BosGru_v2.0) using the HiSAT2 software (Kim et al., 2015). All the samples were assessed by genomic and gene alignment. After obtaining the comparison result bam file, the reads on the comparison gene were assembled using the StringTie software (Pertea et al., 2015), and every single transcript assembled by each sample was fused and spliced into a merged transcript.

### LncRNAs Identification and LncRNAs Target Gene Prediction

We performed the following steps to obtain the potential lncRNA candidates for subsequent analysis: (1) The Cuffcompare software (Ghosh and Chan, 2016) was used to compare the merged transcripts with the reference transcripts one by one, and the transcripts marked with “i,” “u,” “x,” and “o” were retained after clarifying the position class of the remaining transcripts. (2) Transcripts with length >200 nt and exon number ≥2 were obtained. (3) The above transcripts were analyzed about the coding ability by CPC2 (Kang et al., 2017), CNCI (Sun et al., 2013), PLEK (Li et al., 2014), and Pfam (Sonnhammer et al., 1998) software to remove the transcripts with coding potential and obtain potential lncRNA candidates.

*Cis*-acting and *trans*-acting modes are two main ways to predict the targets of lncRNAs (Mercer et al., 2009). We calculated the locations of the paired lncRNAs and mRNAs for the *cis*-acting prediction. The lncRNA with no nearest protein-coding gene within 100 kb upstream or downstream was excluded in subsequent analysis. For *trans*-acting regulatory mode, the LncTar was used to calculate the free energy between them to predict the regulatory targets, since the expression of lncRNA is determined to be independent of the location of mRNA.

### CircRNA Identification

We used the CIRI software and predicted the circRNA based on the BWA software (Li and Durbin, 2009; Gao et al., 2015). Since it is an authoritative software, it has the characteristics of high sensitivity and multiple screening for reducing false positives. Firstly, we aligned the clean reads to the reference genome to obtain the SAM file using the BWA software. Then, the CIRI software was used to scan for PCC signals (paired chiastic clipping signals), and circRNA sequences were predicted based on junction reads and GT-AG cleavage signals. The expressional levels of circRNAs were quantified by the RPM algorithm.

### Differential Expression Genes and Pathway Analysis

The expression levels of the mRNAs and lncRNAs were calculated through the fragments per kilobase of transcript per million reads mapped (FPKM) value (Liu et al., 2018) using the Cuffdiff program. The DESeq software (Anders and Huber, 2010) was used to standardize the counts of each sample and calculate the fold change (FC). A negative binomial distribution test (NB) was used to test the difference significance of counts. The differentially expressed mRNAs (DEMs), lncRNAs (DELs), and circRNAs (DECs) were screened according to the results of |log 2 FC| >1 and *p* < 0.05 eventually.

To further understand gene function, Gene Ontology (GO, http://www.geneontology.org) terms and the Kyoto Encyclopedia of Genes and Genomes (KEGG, https://www.genome.jp/kegg/) were used for enrichment analysis of functional pathways. Each GO and KEGG enrichment term was confirmed by Hypergeometric Distribution Test. Then, the *p*-value was corrected by Benjamini and Hochberg multiple tests. The enrichment with *p*-value lower than 0.05 was considered significant.

### Construction of ceRNA Network

To acquire a better understanding of the interactions of the mRNAs, lncRNAs, circRNAs, and miRNAs, a lncRNA–circRNA–miRNA–mRNA regulatory network was constructed based on the ceRNA hypothesis (Salmena et al., 2011). MiRanda (John et al., 2004) was used to predict the pairs of miRNA–lncRNA, miRNA–mRNA, and miRNA–circRNA. The Spearman correlation coefficient (SCC) was used to evaluate the pairwise correlations of miRNA–lncRNA, miRNA–mRNA, and miRNA–circRNA; the value greater than 0.8 was considered relevant for constructing the network and *p* < 0.05 was regarded as being statistically significant. The Cytoscape software (version 3.5.1) was used to display the results visually.

### Quantitative Real-Time PCR for Validating Gene Expression

The RNA for verifying the gene expression was the same as RNA used in the above Illumina sequencing. The RNA was reverse transcribed into cDNA by HiScript^®^ II 1st Strand cDNA Synthesis Kit (Vazyme, Nanjing, Jiangsu, China). Glyceraldehyde-3-phosphate dehydrogenase (*GAPDH*), as endogenous control, was used to normalize target gene expression. The total 20-μl reaction system of qPCR included 10 ng of cDNA, 10 μl of SYBR Premix Ex Taq II (TaKaRa, Dalian, China), each 1 μl of forward primer and reverse primer, and 7 μl of ddH_2_O. The RT-qPCR conditions included the preincubation at 95°C for the 30 s, 45 cycles of 10 s at 95°C and 60 s at 59°C, then ended at 72°C for 30 s. Each experiment was repeated three times, and the results of relative RNA expression were calculated according to the cycle threshold (Ct) value 2^–ΔΔCt^ (Livak and Schmittgen, 2001).

## Results

### Comparison of Production Performance

Analysis of the measurement results showed that the cattle-yaks had a significant improvement in production performance compared to the yaks in each age stage (*p* < 0.001). As can be seen from Table 1, cattle-yaks were stronger and taller at birth than yaks. In terms of body weight, at birth, the cattle-yak increased by about 22.60% compared to the yaks, and the body length, height, and chest girth of cattle-yaks were also increased by 17.62%, 11.06%, and 8.92%, respectively. From birth to 6 months old, the cattle-yak showed obvious heterosis with varying degrees of improvement in various indicators.

**TABLE 1:** The production performance between cattle-yaks and yaks.

Items	Birth (Mean ± SE)			3 months (Mean ± SE)			6 months (Mean ± SE)		
	Yaks	Cattle-yaks	*p*-Value	Yaks	Cattle-yaks	*p*-Value	Yaks	Cattle-yaks	*p*-Value
Body weight (kg)	16.37 ± 0.23	20.70 ± 0.41	<0.001	37.56 ± 0.51	48.21 ± 0.62	<0.001	81.40 ± 1.31	90.76 ± 0.81	<0.001
Body height (cm)	57.23 ± 0.87	63.56 ± 0.56	<0.001	73.73 ± 0.98	84.80 ± 0.95	<0.001	85.43 ± 1.12	91.73 ± 0.86	<0.001
Body length (cm)	50.50 ± 1.10	59.40 ± 0.55	<0.001	75.63 ± 0.72	87.27 ± 0.78	<0.001	89.90 ± 0.77	98.07 ± 1.04	<0.001
Chest girth (cm)	58.63 ± 0.79	63.86 ± 0.54	<0.001	84.46 ± 0.60	97.23 ± 0.68	<0.001	112.83 ± 1.23	120.77 ± 1.20	<0.001

Note: SE: standard error.

### The Prediction and Characteristic of LncRNAs and CircRNAs

After filtering out the low-quality and supernumerary reads, we obtained a total of 310,089,232 and 308,742,564 clean reads with greater than 93.77% of Q30 from the LD muscles of cattle-yaks and yaks, respectively. As shown in Supplementary Table S1, the successful alignment of reads to the reference genome was approximately over 94.55%.

**FIGURE 1:**
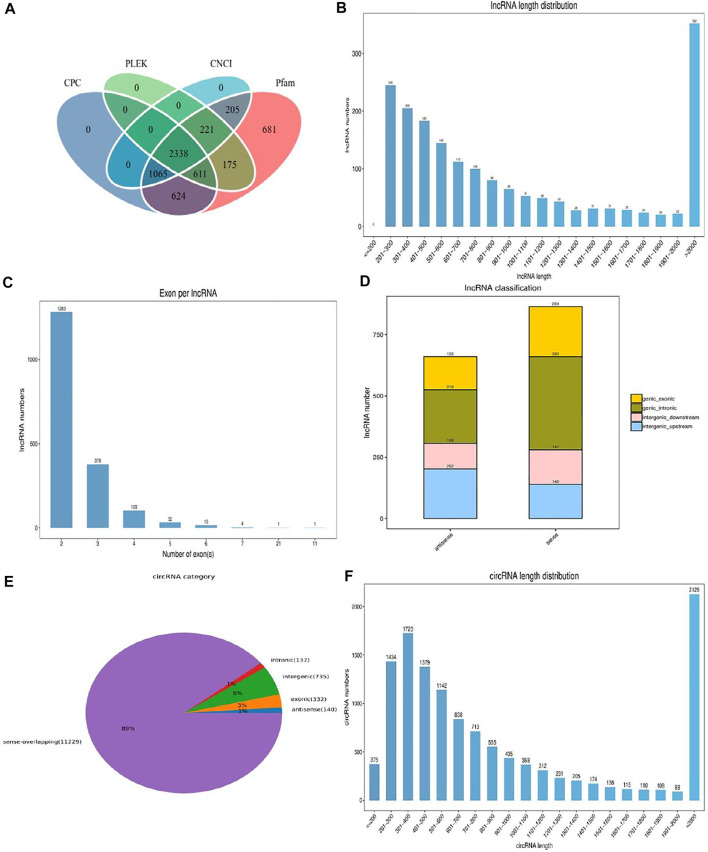
The description of identified lncRNAs and circRNAs. **(A)** Screening of the candidate lncRNAs in *longissimus dorsi* muscle. **(B)** The length distribution of the novel lncRNAs. **(C)** Exon number distribution of novel lncRNAs. **(D)** The classification of novel lncRNAs. **(E)** The structure type pie chart of circRNAs. **(F)** The length distribution of circRNAs.

After rigorous screening and filtration, we detected a large number of lncRNAs and cirRNAs (Figures 1A,E). Among them, 1,817 lncRNAs with an average length of 1,449 bp were discovered as novel lncRNAs (Figure 1B). The largest proportion of lncRNAs was over 2,000 bp (19.37%) and the proportion of lncRNA containing two exons was about 70.61% (Figure 1C). The total lncRNAs of 135 exonic antisense, 219 intronic antisense, 105 intergenic downstream antisense, 202 intergenic upstream, 204 exonic sense, 380 intronic sense, 141 intergenic downstream sense and 140 intergenic up-stream sense were identified in our results (Figure 1D). The average length of detected circRNAs was 3,250 bp and the length of the most circRNAs was over 2,000 bp (16.92%) (Figure 1F). As evident in Figure 1E, the circRNAs of sense-overlapping accounts for 89% of the total. The number of circRNAs located in the exonic and intronic was 332 and 137, respectively, while the antisense-overlapping circRNAs occupied about 1%.

### Differentially Expressed mRNAs, lncRNAs, and circRNAs Between CY and Y Groups

**FIGURE 2:**
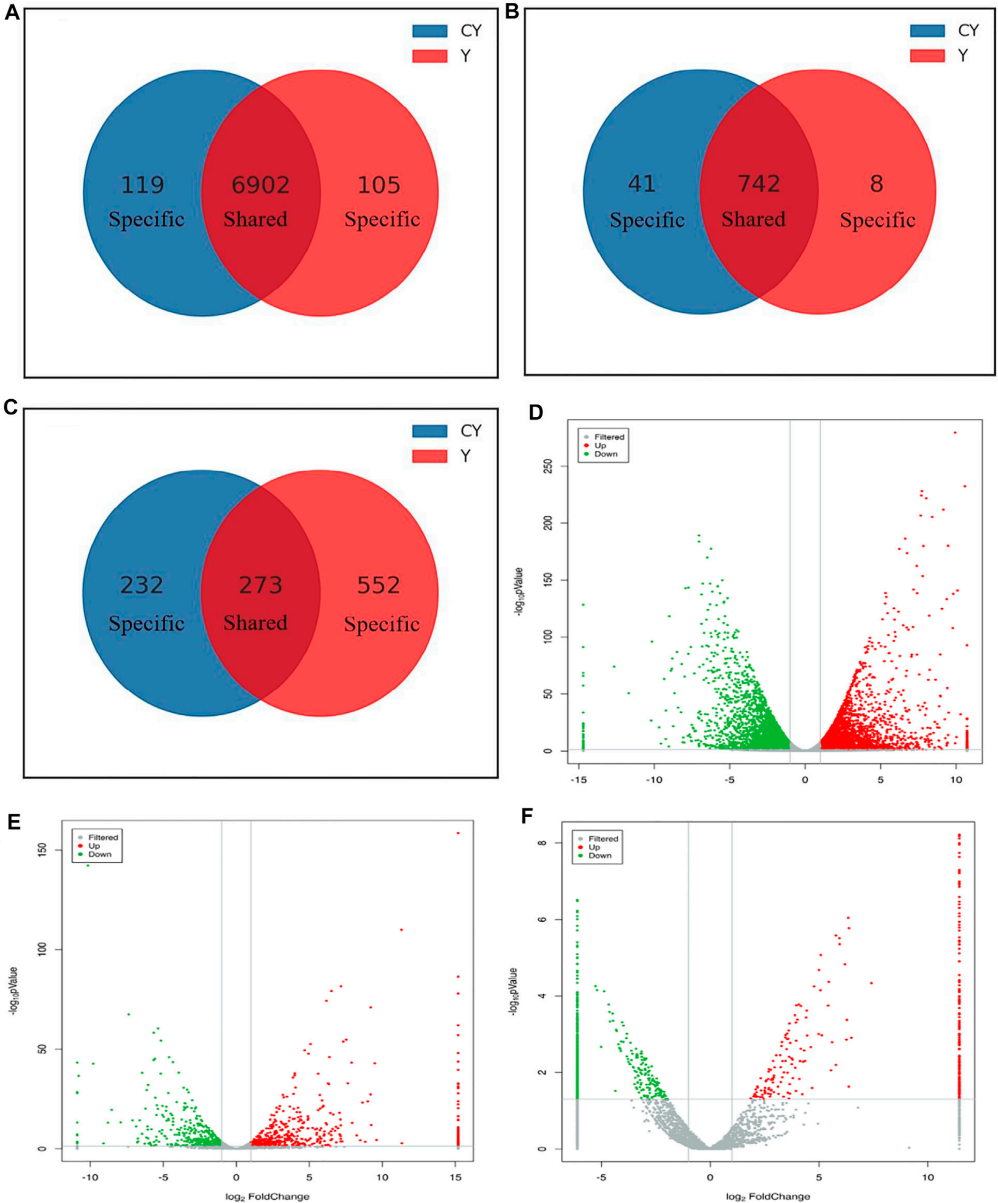
Comparative analysis of the differentially expressed mRNAs and ncRNAs between cattle-yaks and yaks. The specific and shared mRNAs **(A)**, lncRNAs **(B)**, and circRNAs **(C)** between the two groups. The analysis of differentially expressed mRNAs **(D)**, lncRNAs **(E)**, and circRNAs **(F)** between two groups. Note: The red and green points represented upregulated and downregulated mRNAs, lncRNAs, and circRNAs, respectively. The gray points represented no significant differences. The gray vertical lines showed |log2FC| = 1, and the gray horizontal lines showed *p* = 0.05.

The study identified 7,126 mRNAs, 791 lncRNAs, and 1,057 circRNAs to have significant differential expressions (Supplementary Tables S2–S4). There were 6902 DE mRNAs, 742 DE lncRNAs, and 273 DE circRNAs, respectively, which were detected in the cattle-yaks and yaks (Figures 2A–C). However, 119 mRNAs, 41 lncRNAs, and 232 circRNAs were detected to express only in the CY group. Other 105 mRNAs, 8 lncRNAs, and 552 circRNAs were identified only in the Y group. To find the overall distribution of differential expression, the volcano plot was drawn based on the results of the differential expression. Compared with the yaks of the control group, we found 3,563 upregulated mRNAs, 455 upregulated lncRNAs, and 353 upregulated circRNAs in the cattle-yaks. Meanwhile, there were 3,563 downregulated mRNAs, 336 downregulated lncRNAs, and 704 downregulated circRNAs, respectively, in the cattle-yaks (Figures 2D–F).

### Functional Analysis

**FIGURE 3:**
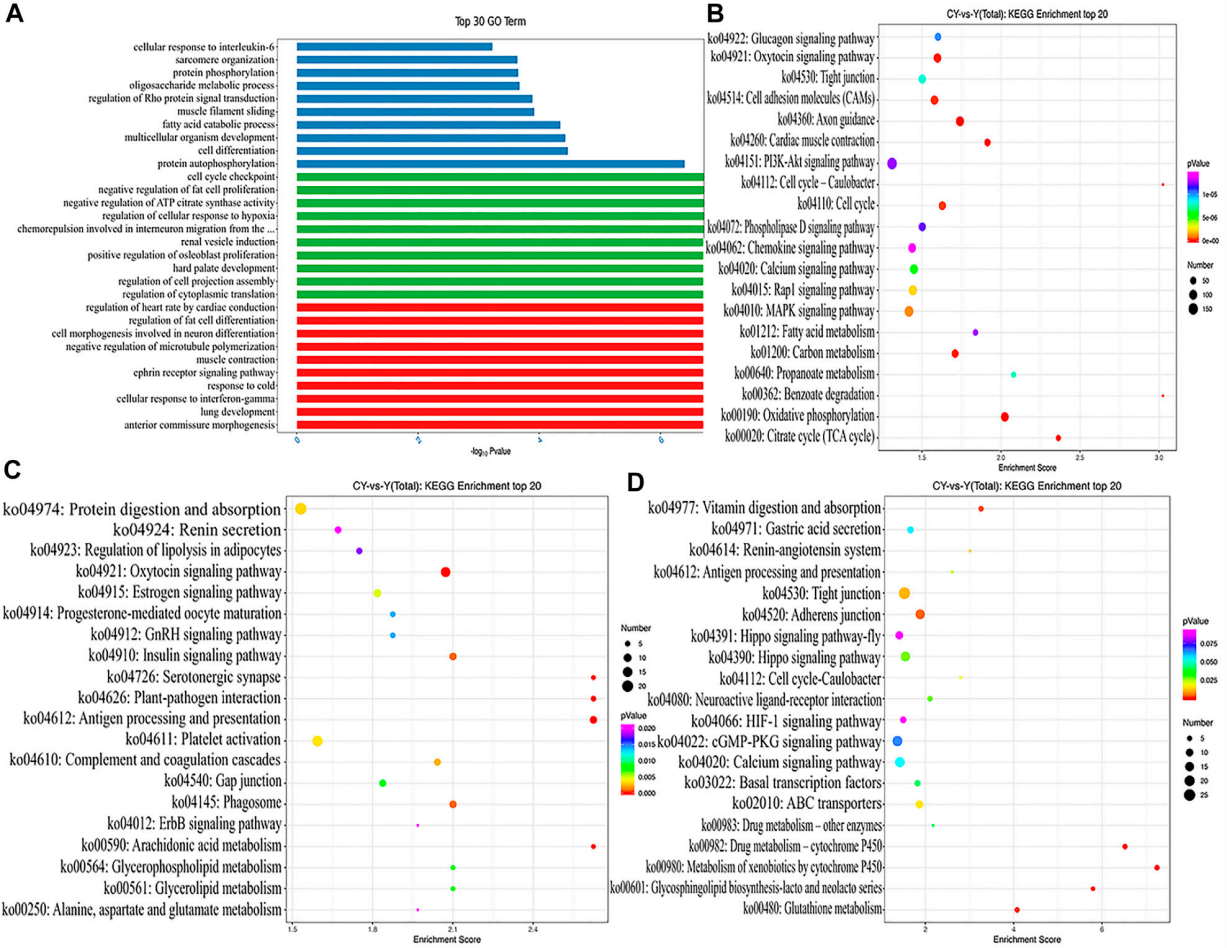
**(A)** GO analysis with the top 10 enrichment biological processes for DEMs, DELs targets, and DECs host genes between cattle-yaks and yaks. KEGG analysis with the top 20 KEGG enrichment pathways for DEMs **(B)**, DELs target genes **(C)**, and DECs host genes **(D)** between the cattle-yaks and yaks.

GO analysis described the molecular functions performed by the DE ncRNAs, the cellular environment in which they were located, and the biological processes involved. We respectively selected the top 10 biological processes with the most significant enrichment of mRNAs, lncRNAs, and circRNAs (Figure 3A). Obviously, the DEMs were mainly enriched in regulation of osteoblast proliferation, negative regulation of fat cell proliferation, and regulation of cellular response to hypoxia. The targeted genes of DELs were most enriched in terms involving regulation of fat cell differentiation, ephrin receptor signaling pathway, and muscle contraction. Protein autophosphorylation, fatty acid catabolic process, and regulation of Rho protein signal transduction were the most significant enrichment terms for the sourced genes for DECs. As evident from Figures 3B–D, we conducted the KEGG analysis using KEGG public pathway database and draw the augmented scatter diagram of the selected target genes. Between cattle-yaks and yaks, the DEMs were enriched in the PI3K−Akt signaling pathway, MAPK signaling pathway, Fatty acid metabolism, Citrate cycle, etc. (Figure 3B). The DEL adjacent genes were significantly related to the protein digestion and absorption, GnRH signaling pathway, alanine, aspartate and glutamate metabolism, and so on (Figure 3C). The host genes of DECs were significantly associated with some pathways, such as those related to vitamin digestion, ABC transporters, cGMP−PKG signaling pathway, etc. (Figure 3D). Interestingly, some DEMs and the host genes of DECs were found to be enriched in the same pathways (i.e., hippo signaling pathway, cell cycle-caulobacter, and calcium signaling pathway), and some DEM and DEL adjacent genes were enriched in the same oxytocin signaling pathways.

**FIGURE 4:**
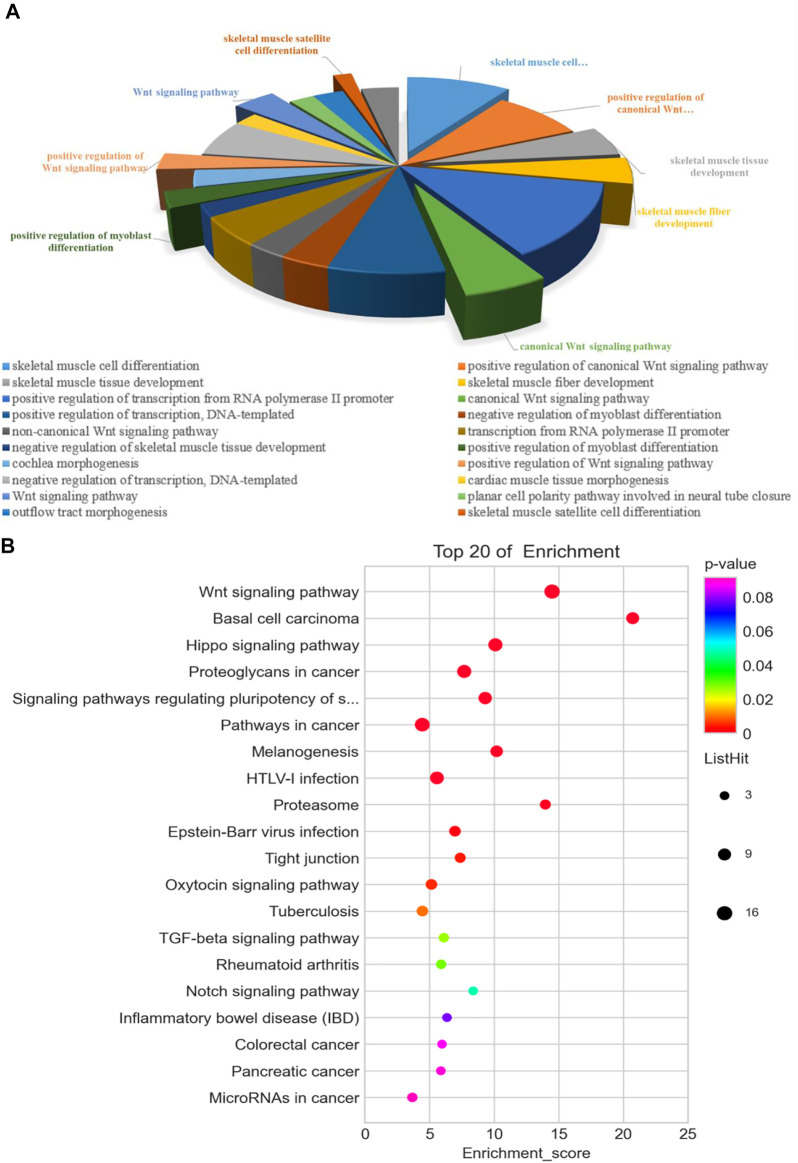
Functional analysis of 117 differentially expressed genes associated with muscle development and fatness between the cattle-yak and yak. GO **(A)** and KEGG **(B)** analysis.

In order to further explore the DEMs related to muscle growth and fatness, we screened out the related genes. These 117 DEMs related to muscle development and fat deposition are shown in Supplementary Table S5. The functional predictions of GO in DEMs (Figure 4A) mainly focused on some terms of skeletal muscle development, muscle cell differentiation, and regulation of canonical Wnt signaling pathway, including positive regulation of myoblast differentiation and skeletal muscle fiber development. According to the KEGG pathway analysis (Figure 4B), some pathways were enriched significantly by these DEMs, such as Hippo signaling pathway, regulating many biological processes involved in proliferation, survival and differentiation of cell, organ size, and tissue homeostasis. In addition, some DEMs enriched significantly in Notch signaling pathway that can regulate the differentiation and development of cells, tissues, and organs through the interaction between the adjacent cells.

### Construction of the ceRNA Coregulatory Network

**FIGURE 5:**
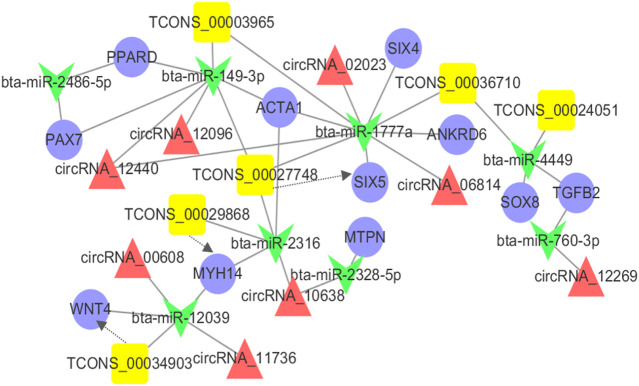
The ceRNA co-regulation network. The blue circle, yellow box, red triangle and green V-type represented the differentially expressed mRNAs, lncRNAs, circRNAs, and miRNA, respectively. The solid line indicated the co-regulation between miRNAs and other transcripts. The dotted line indicated the co-regulation between the lncRNAs and mRNAs.

Previous studies have shown that mRNAs, lncRNAs, and circRNAs may regulate gene function through miRNAs as ceRNAs in different processes (Salmena et al., 2011; Yu et al., 2019), indicating that ceRNAs and their miRNAs may work in concert with each other. Combined with the DEMs, DELs, and DECs related to muscle development and co-differentially expressed, we constructed the integrated ceRNA network. This ceRNA network contained 11 DEMs, 6 DELs, 8 DECs, and 33 relationships (Figure 5). Tcons-00034903 and bta-miR-2039 have a shared target gene *WNT4*. Similarly, we also found the same results in bta-miR-2316-Tcons-00029868-*MYH4* and bta-miR-1777a-Tcons-00027748-*SIX5*. This ceRNA network might provide valuable information for the development of the *longissimus dorsi* in cattle-yaks and yaks.

### Real-Time Quantitative PCR Validation of Sequencing Data

**FIGURE 6:**
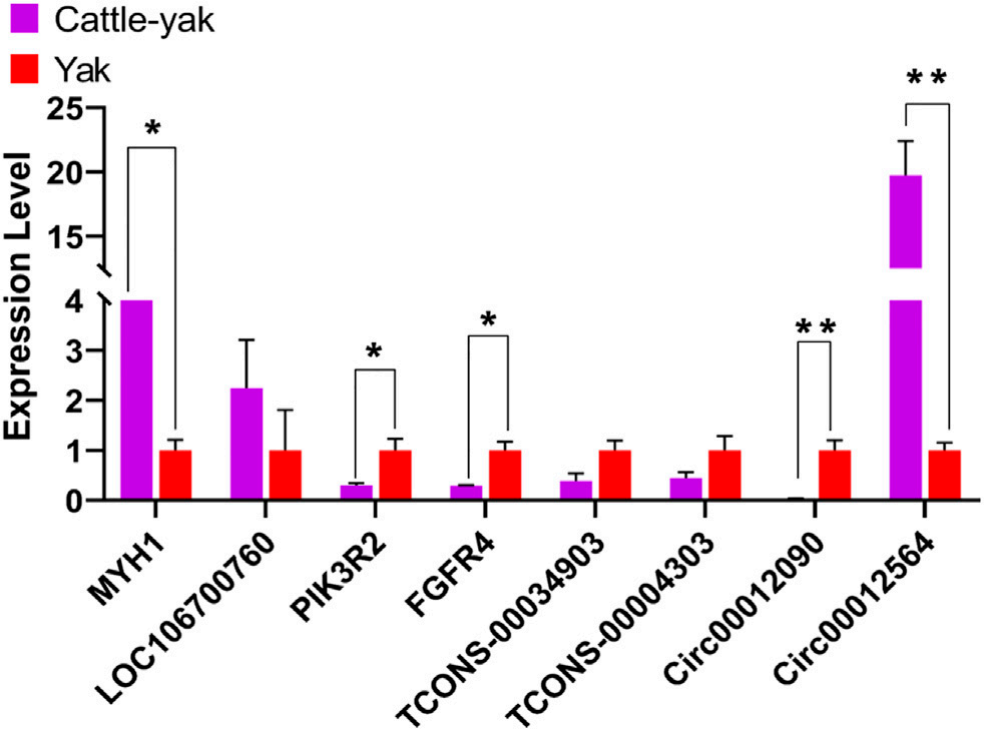
The results of the real-time quantitative PCR validation of the expression level. ** indicates *p* < 0.01, * indicates *p* < 0.05. The data represented the mean ± SEM from three biological replicates, and each measurement was repeated 3 times.

Four mRNAs (*MYH14*, *LOC106700760*, *PIK3R2*, and *FGFR4*), 2 lncRNAs (Tcons-00034903 and Tcons-00004303), and 2 circRNAs (circ00012096 and circ00012564) were selected randomly for verifying the sequencing results through real-time quantitative PCR (Figure 6). The primers were designed using the Primer-BLAST web tool from the National Center for Biotechnology Information (NCBI) (Supplementary Table S6). Their expression patterns were highly consistent with the sequencing results, indicating that the gene expression profiles obtained in this study had high repeatability and reliability.

## Discussion

Muscle growth is a complex economic trait owing to various physiological and biochemical indices. It concomitantly involves many gene expressions and regulations. To date, people have a great demand for high-quality meat and nutrition with improving living standards; it is an urgent problem for improving the performance of yaks. Cattle-yak, as hybrid offspring of yak (♀) and cattle (♂), has been found to have significant improvement in the production performance (Guo et al., 2019; Dingkao et al., 2020; Luo et al., 2020; Jiang et al., 2021). However, the economic benefit of cattle-yaks has been ignored for a long time. The variations in the genes and proteins in the muscle structure are presumed generally to be affected by production performance (Joo et al., 1999); this study is the first time to systematically explore the differences of growth and development between cattle-yaks and yaks based on transcriptomics. To study the regulatory mechanism of growth and muscle development of cattle-yaks and yaks in a better way, we performed transcriptome analysis of the *longissimus dorsi* muscle. It was also the first time to compare the differences in the expression profiles of mRNA, lncRNA, and circRNA between the cattle-yaks and yaks, so as to determine the key factors involved in muscle growth and development. We measured the phenotypic data strictly of 30 cattle-yaks and yaks at three age groups of growth using the standard method of measurement, and the results obtained were similar to the previous studies; in each period of cattle-yaks with the same feeding conditions, production performance indexes of cattle-yaks were significantly higher than those of yaks (*p* < 0.001). We speculated that these differences may be due to genetic factors rather than the effects of feeding management on these traits. Therefore, the study on the mechanism regulating muscle growth and development in hybrid yaks and yak breeds can better help in enhancing the production performance of the yaks, providing help for exploring the regulatory mechanism of muscle development in mammals.

Typically, the growth and development of muscles are regulated by the core genes and signal transduction pathways (Myers et al., 2006; Ayuso et al., 2015). Compared to the yak group, a total of 7,126 DEMs, 791 DELs, and 1,057 DECs were identified in the cattle-yak group. Subsequently, the GO and KEGG pathway enrichment analysis revealed some important DEMs related to muscle growth and fat deposition, which was consistent with the results obtained after measuring the production performance, indicating that the production performance of the cattle-yak was better than that of the yak (Tumennasan et al., 1997). In addition, some DEMs, DELs, and DECs related to the immune system have reflected the adaptation of the yak to the high-altitude environment, and the adaptation of the yaks to the cold and high-altitude environment was well-preserved by the cattle-yaks. In succession, we focused on some DEMs related to the production performance of cattle-yaks and yaks. A total of 117 DEMs were identified that were related to the differentiation and proliferation of myoblasts, AMPK signaling pathway, MAPK signaling pathway, PI3K–Akt signaling pathway, etc. (Neri et al., 2002; Anderson, 2006; Gehart et al., 2010; Thomson, 2018). Among these DEMs, some have been found to have known functions in muscle growth and development. For example, myostatin (*MSTN*), as a member of the TGF-β superfamily, has a proven role as a growth differentiation factor (Kollias and McDermott, 2008; Li et al., 2008) playing an important role in mice (McPherron et al., 1997), cattle (McPherron and Lee, 1997), and humans (Schuelke et al., 2004) by negatively regulating the growth and differentiation of myoblasts, as well as other mammals *via* controlling both the activation and proliferation of the satellite cells (skeletal muscle stem cells) (McCroskery et al., 2003). The expression level of the *MSTN* gene in our study was found to be downregulated in the cattle-yaks; hence, the expression of this gene was considered to be consistent with its function. This also provided new evidence for the function of the *MSTN* gene in *longissimus dorsi* of cattle-yaks and the high conservation across species. Myogenin (*MYOG*), including myogenic factor 6 (*MYF6*), is a regulatory factor in the family of Myogenic regulatory factors (*MRFs*), which is mainly involved in the fusion and differentiation of myoblasts (CHARGÉ and RUDNICKI, 2004; Buckingham and Vincent, 2009). Interestingly, the paired box transcription factor 7 (PAX7) gene and the MYOG gene were found to be downregulated, but the MYF6 expression was upregulated in the cattle-yaks. Previous studies have shown that overexpression of gene *PAX7* can induce *MYOG* expression to inhibit myogenesis and prevent the differentiation of the muscle cell (Chen et al., 2010; Dey et al., 2011). Therefore, this also justified the downregulation of both *PAX7* and *MYOG* gene expression in cattle-yaks with better production performance than yaks. Additionally, some genes, such as actin alpha 1 skeletal muscle (*ACTA1*) and actin alpha cardiac muscle 1 (*ACTC1*), also play a key role in the differentiation and fusion of muscle cells, and have a positive impact on the myogenesis of the skeletal muscles (Ciecierska et al., 2020). Although these studies have helped in predicting the functions and accuracy of the key genes, the other functional DEMs related to muscle growth and development still need to be further studied on their expression regulation in *longissimus dorsi* muscle.

The main non-coding RNAs, the lncRNAs and circRNAs, are receiving more and more attention, as they can participate in the regulation of various biological processes in different ways (Liu et al., 2017; Marchese et al., 2017; Wang et al., 2019a). The identified DELs and DECs in this study were involved in regulating the promotion of muscle growth and development. LncRNA can exert its important action through various biological and pathological processes by demonstrating trans, cis, and antisense effects. Our results showed abundant differentially expressed lncRNAs in the skeletal muscle of cattle-yak and yak, suggesting that the lncRNAs may not be the exclusive by-products of mRNA in the cattle-yak but have specific roles. The long non-coding RNA acts differently in the nucleus and cytoplasm due to their different locations, where they are involved in the muscle development regulation on both embryonic and growing stages. According to the KEGG analysis, the differentially expressed lncRNAs and circRNAs were identified to functionally relate to some hormone regulation, myoblast proliferation, and metabolic pathways. CircRNAs being another type of non-coding RNA also act as an important regulatory role in skeletal muscle growth and development (Greco et al., 2018). Multiple studies have confirmed that abundant circRNAs exist in the skeletal muscle and their expression levels change dynamically during the process of myoblast differentiation (Legnini et al., 2017). Notably, we found that many DECs host genes are enriched in the HIF-1 signaling pathway in the KEGG analysis. HIF-1 signaling pathway is the core signaling pathway induced by hypoxia involved in regulating the proliferation and differentiation of myoblasts upon hypoxic conditions (Ogilvie et al., 2000). HIF-1 pathway can upregulate its target genes, some of which involve the regulation of proliferation and differentiation of myoblasts. These DEC host genes were found to be significantly enriched in the HIF-1 signaling pathway, indicating a close relationship with the regulation of muscle growth and development under high-altitude and hypoxic environments. The regulatory mechanism of these circRNAs and their host genes on muscle development hence deserves further studies. Furthermore, our studies also indicated some lncRNAs and circRNAs to be specifically or mainly expressed in the *longissimus dorsi* muscle of cattle-yaks (e.g., Tcons-00005361, Tcons-00037918, and circRNA-02529), indicating that these ncRNAs were generated on purpose to have specific effects in the muscle development of the cattle-yaks.

In recent years, extensive studies on the function of miRNAs have provided a new theory named competing for endogenous RNA (ceRNA). To understand the process of development of the skeletal muscle, a ceRNA regulatory network was constructed based on the combination with the DEMs, DELs, and DECs related to muscle development and were expressed co-differentially. The results showed that 11 DEMs, 6 DELs, and 8 DECs cross-talked with another through 8 differential expressions of the microRNAs. This also indicated that the development of the *longissimus dorsi* muscle in cattle-yak was a complex regulation process of a balanced level of gene expression under a high-altitude and hypoxic environment. As reported, peroxisome proliferator-activated receptor delta (*PPARD*) acted as a vital regulator in adipogenesis and lipid metabolism (Kim et al., 2006; Chen and Yang, 2014). Ankyrin repeat domain 6 (*ANKRD6*) also played a role in regulating crucial events in developing vertebrates and invertebrates (Schwarz-Romond et al., 2002; Moeller et al., 2006). Therefore, we speculated that these ncRNAs might also contribute to muscle development by indirectly regulating the gene expression of *PPARD* and *ANKRD6*. Not only that, we observed three important ceRNA subnetworks from the ceRNA network, showing that TCONS-00024051 and its target *SOX8* “talked” to each other through the same bta-miR-1777a response element, while TCONS-00034903 and its target *WNT4*, TCONS-00029868 and its target *ACTA1* “talked” to each other through bta-miR-12039 and bta-miR-4449 response elements, respectively. Therefore, we speculated that these three subnetworks may be crucially associated and function in regulating muscle development. Notably, as a member of the “unconventional” non-muscle myosin II family of molecular motors, myosin heavy chain 14 (*MYH14*) gene has been identified as a key regulator of muscle fiber type (van Rooij et al., 2009; Bell et al., 2010). In our results, *MYH14* and Tcons-00029868 were both upregulated in *longissimus dorsi* muscle, which indicates that this lncRNA may have a *cis*-regulatory relationship with *MYH14* gene. From our results, these DEMs, DELs, and DECs were not only involved in the process of muscle development, but might also be involved in lipogenesis as ceRNAs.

## Conclusion

In conclusion, we compared the production performance of cattle-yaks and yaks, it was also the first time to compare the expressional features of mRNAs, lncRNAs, and circRNAs in the *longissimus dorsi* tissue of cattle-yaks and yaks. In our results, the abundant mRNAs and ncRNAs were identified, some of which were specifically expressed in cattle-yaks. According to the bioinformatics analyses, the ncRNAs were found to be not only connected with the myoblast differentiation and proliferation, skeletal development, and signaling pathway of muscle growth, but be also useful as ceRNAs of important transcription factors (such as *SOX8* and *PPARD*). In addition, the ceRNA network (11 DEMs, 6 DELs, and 8 DECs) that we constructed may have the considerable effects on regulation of muscle growth. This study provided new insights into the genetic basis of muscle growth and laid the foundation for further study of the role of these ncRNAs in regulating muscle growth.

## Data Availability

The datasets presented in this study can be found in online repositories. The names of the repository/repositories and accession number(s) can be found below: https://www.ncbi.nlm.nih.gov/, PRJNA753699.
